# Diagnostic Accuracy of the RDW for Predicting Death in COVID-19

**DOI:** 10.3390/medicina58050613

**Published:** 2022-04-28

**Authors:** Eduardo Guaní-Guerra, Brenda Torres-Murillo, Carolina Muñoz-Corona, José Carlos Rodríguez-Jiménez, Alejandro E. Macías, David A. Scavo-Montes, Jose A. Alvarez

**Affiliations:** 1 Department of Research, Hospital Regional de Alta Especialidad del Bajío, San Carlos La Roncha, León P.C. 37660, Guanajuato, Mexico; eduardoguani@yahoo.com.mx; 2 Department of Medicine, University of Guanajuato, San Carlos La Roncha, León P.C. 37660, Guanajuato, Mexico; bren20.to@gmail.com; 3 General Directorate of Quality and Health Education, Ministry of Health, Mexico City P.C. 11410, Mexico; itzatcarolina07@gmail.com; 4 Department of Internal Medicine, Hospital Regional de Alta Especialidad del Bajío, San Carlos La Roncha, León P.C. 37660, Guanajuato, Mexico; jc.rodriguezjmz@gmail.com; 5 Microbiology Laboratory, Department of Medicine and Nutrition, University of Guanajuato, León P.C. 37000, Guanajuato, Mexico; aaeemmhh@yahoo.com; 6 Hospital Estatal de Atención COVID-19, León P.C. 37000, Guanajuato, Mexico; davidscavomontes@gmail.com

**Keywords:** red blood cell distribution width (RDW), COVID-19, SARS-CoV-2, mortality, risk

## Abstract

*Background and Objectives*: An association between high red blood cell distribution width (RDW) and mortality has been found in several diseases, including infection and sepsis. Some studies have aimed at determining the association of elevated RDW with adverse prognosis in COVID-19, but its usefulness has not been well established. The objective of this study was to determine the accuracy of the RDW, measured at hospital admission and discharge, for predicting death in patients with COVID-19. *Materials andMethods*: An observational, retrospective, longitudinal, and analytical study was conducted in two different COVID-19 reference centers in the state of Guanajuato, Mexico. A total of 323 patients hospitalized by COVID-19 were included. *Results:* We found higher RDW levels at the time of hospital admission in the non-survivors group compared to levels in survivors (median = 13.6 vs. 13.0, *p* < 0.001). Final RDW levels were even higher in the deceased group when compared with those of survivors (median = 14.6 [IQR, 12.67–15.6] vs. 12.9 [IQR, 12.2–13.5], *p* < 0.001). For patients who died, an RDW > 14.5% was more common at the time of death than for patients who survived at the time of discharge (81 vs. 13 patients, *p* < 0.001; RR = 2.3, 95% CI 1.89–2.81). *Conclusions*: The RDW is an accessible and economical parameter that, together with other characteristics of the presentation and evolution of patients with COVID-19, can be helpful in determining the prognosis. An RDW that increases during hospitalization could be a more important mortality predictor than the RDW at hospital admission.

## 1. Introduction

Although the number of new COVID-19 cases and deaths continues to decrease worldwide, death incidences remain high and significant increases have been reported in many countries. By 13 March 2022, the cumulative number of confirmed COVID-19 deaths around the world was over 6 million [1]. It is already well known that COVID-19 not only causes acute disease, but also generates persistence of symptoms, including chronic fatigue syndrome and myalgic encephalomyelitis [2]. Thus, the identification of clinical and laboratory factors, as well as COVID-19 biological phenotypes predictive of clinical deterioration and prognosis, is a top research priority in the ongoing COVID-19 pandemic [3,4].

Evidence supports the usefulness of biomarkers such as C-reactive protein, troponin, and D-dimer in predicting mortality, disease severity, or thrombotic complications among patients hospitalized for COVID-19 [5,6]. However, they could be expensive, not available, and time consuming in emergent economies.

Red blood cell distribution width (RDW), calculated by dividing the standard deviation of corpuscular volume by the mean corpuscular volume, is a parameter of the hemogram used in the differential diagnosis of anemia and involves the variability in form and size of red blood cells in the subject [7]. Previous studies have found evidence, in some specific conditions, that RDW elevation is caused by the delayed clearance of older red blood cells (RBCs) [8,9]; an association between high RDW and mortality has been found in patients with coronary disease, liver disease, pancreatitis, ischemic stroke, and sepsis [10,11,12,13,14]. In addition, some studies have been aimed at determining the association of elevated RDW with an adverse prognosis in COVID-19 [15,16,17,18,19,20,21]; its usefulness, however, has not been well established. Thus, the aim of this study was to determine the accuracy of the RDW, measured at hospital admission and discharge, for predicting death in patients with COVID-19.

## 2. Materials and Methods

We conducted an observational, retrospective, longitudinal, and analytical study in two different COVID-19 reference centers (Regional Hospital of High Specialty at Bajio and COVID-19 State Hospital) in the state of Guanajuato, Mexico. Epidemiological, clinical, and laboratory data, such as RDW, absolute leucocyte count, D-dimer level, and C-reactive protein, were obtained by reviewing clinical files.

Patients were excluded from the study if they were aged less than 18 years. COVID-19 diagnosis was confirmed by real-time reverse transcriptase-polymerase chain reaction for SARS-CoV-2 during hospitalization. The complete blood count (CBC) with differential was performed using a CELL-DYN Ruby Hematology Analyzer (Abbott Laboratories) at these two COVID-19 reference centers. An RDW cutoff point of 14.5% was considered as it has been reported previously in another study [19].

The study was approved by the ethics and research committee of the Regional Hospital of High Specialty at Bajio, Mexico (approval number CEI-30-2021), and was authorized for a waiver of patient informed consent because the study involved data collected for non-research purposes and involved minimal or null risk. Quantitative variables were described using median with interquartile range (IQR), and mean ± standard deviation (SD). Categorical data were described using absolute and relative frequencies. Statistical differences between study groups (survivors vs. non-survivors) were calculated using a 2-sided *t*-test for means, Mann–Whitney U test for quantitative variables with non-parametric distribution, and χ^2^ test for proportion comparison test for incidence rates (%). Relative risks were calculated for qualitative dichotomous variables. Statistical analysis was performed using the Internet-nested statistical suite VassarStats.net. A receiver operating characteristic (ROC) curve was made to determine the area under the curve (AUC), sensitivity, specificity, and the cutoff for initial and final RDW as predictors of death. Logistic regression analysis was performed to identify, by hierarchical forward selection, the qualitative and quantitative variables associated with patient death. Seven likelihood iterations were carried out to discard non-significant variables until modeling of the relationship between the independent and dependent variables was found. The AUC of the ROC curve (AUC, as a measure of goodness of fit for binary outcomes in a logistic regression model) was calculated. Higher values of AUC identify a better discriminating ability of the model between a true positive and a false positive value. The ROC and diagnostic analysis for initial and final RDW, and the logistic regression analysis were performed with NCSS 12.0.2 Statistical Software (NCSS, LLC, Kaysville, UT, USA, 2018). All statistical tests used were two-sided, and *p* < 0.05 was considered statistically significant.

## 3. Results

We included 323 study patients, 211 males (65.3%) and 112 females (34.7%); with a mean age of 55.98 ± 14.16 years. A total of 248 patients (76.8%) were reported with associated comorbidities, the most frequent being diabetes (45.2%), hypertension (44.9%), and overweight (30.3%). Patients requiring invasive mechanical ventilation were reported in 195 (60.4%) cases. Regarding anticoagulant therapy, 255 patients received low molecular weight heparin, and 50 patients received unfractionated heparin (UFH). Hospital-associated infections were observed in 43 (13.3%) study subjects. The median length of hospital stay (LOS) among patients was 10.70 ± 8.42 days (Table 1).

When patients were stratified by mortality, several clinical and biochemical variables denoted an increased risk of death when comparing survivors vs. non-survivors. Among the most relevant were the requirement of mechanical ventilation (RR = 5.7, 95% CI 3.69–8.78), treatment with UFH (RR = 2.59, 95% CI 1.47–4.56), superinfection (RR = 1.47, 95% CI 1.19–1.81), RDW > 14.5% prior to hospital discharge (RR = 2.3, 95% CI 1.89–2.81), and creatinine > 1.3 mol/L at hospital admission (RR = 1.94, 95% CI 1.64–2.28). Moreover, higher RDW levels were reported at the time of hospital admission in the non-survivors group compared to the levels in survivors (median = 13.6 vs. 13.0, *p* < 0.001). Final RDW levels were even higher in the deceased group when compared with survivors (median = 14.6 vs. 12.9, *p* < 0.001). See Table 2.

The RDW values at hospital admission and hospital discharge were compared between COVID-19 survivors and non-survivors. Deceased patients showed an increment in RDW% during hospitalization (initial RDW median of 13.6 [IQR, 12.94–14.4] vs. final RDW median of 14.6 [IQR, 12.67–15.6], *p* < 0.001), while a slight decrease was observed in the survivor’s group (initial RDW median of 13 [IQR, 12.3–13.7] vs. final RDW median of 12.9 [IQR, 12.2–13.5], *p* = 0.005). See Table 3.

Patients with anemia had a higher initial RDW% (median of 14.6; 95% CI, 13.5–16.5) compared to patients without it (median of 13.2; 95% CI, 12.6–13.9) *p* < 0.001, and in relation to final RDW, patients with anemia also had a higher RDW 15.4 (median of 15.4; 95% CI, 13.9–17.45) compared to patients without it (median of 13.5; 95% CI, 12.6–14.6) *p* > 0.001.

The AUC of the ROC curves of the initial and final RDW, as predictors for death, were 0.655 (95% CI, 0.5848–0.7151) and 0.830 (95% CI, 0.7733–0.8727). The cutoff values of the initial and final RDW were ≥13.30 and ≥13.70, respectively. The logistic regression analysis shows a significant model with three variables contributing to the death of the patient, in a hierarchical forward selection mode excluding 12 variables after seven iterations. The regressor formula is Logit(death) = XB when death = 1(yes) − 14.50 + 0.91 * Final RDW − 2.56 * Mechanical ventilation = 2(no) + 0.05 * Age. The model R^2^ is equal to 0.4829 and the AUC for using the formula model to evaluate the relationship between the final RDW, use of mechanical ventilation, and age with death is 0.917. See Table 4.

## 4. Discussion

As previously mentioned, the RDW has been reported as an independent marker of mortality, independently of other underlying clinical conditions [10,11,12,13,14], and in the context of COVID-19 also [15,16,17,18,19,20,21]. The main findings of our study were as follows: Higher RDW% at the time of hospital admission and hospital discharge were observed in non-survivors when compared to survivors. Patients with RDW > 14.5% measured at hospital discharge showed an increased mortality risk. An RDW increase of 1% was observed in non-survivors during hospitalization; on the other hand, the survivors diminished their RDW levels by 0.1%. The best RDW cutoff values for predicting mortality in COVID-19 patients were 13.30 and 13.70 for RDW measured at hospital admission and discharge, respectively. The AUC determined for RDW was higher when it was measured at hospital discharge.

This is consistent with our findings, as we observed higher RDW at the time of hospital admission in the non-survivors group compared to that in survivors (median = 13.6 vs. 13.0, *p* < 0.001). A difference of 0.6 in RDW% between survivors and deceased is slightly lower than that reported in the meta-analysis performed by Lee, J.J. et al., where they found a pooled mean difference of 0.93 [15]. In addition, the RDW median of 13.6 (IQR, 12.95–14.4) observed in our study group of non-survivors is lower than those reported by Foy (15 ± 2.2), Levy (14.23 ± 1.63), and Nicholson (14.84 ± 1.93) in their respective studies [19,22,23]. Such variability suggests that the RDW prognostic values still remain to be determined; however, in a recent study, Wang et al. reported an RDW cutoff value of 12.85% with a sensitivity of 73.9% and specificity of 81.9% (area under the ROC curve of 0.870, 95% CI 0.775–0.952) for predicting the prognosis of severe COVID-19 patients [20]. In our study, to predict mortality, we reported a cutoff value of 13.30 for the initial RDW with a sensitivity of 62.1% and specificity of 64.4% (AUC of the ROC of 0.655, 95% CI 0.585–0.715); but the cutoff value for the final RDW of 13.70 showed better sensitivity (75.8%) and specificity (78.8%) (AUC of the ROC of 0.830, 95% CI 0.773–0.873).

An elevated RDW (>14.5%) on admission was associated with an increased mortality risk in patients in other studies. For example, Foy et al. observed an RR of 2.73, and Soni/Gopalakrishnan reported a hazard ratio (HR) of 1.84 (95% CI 1.20–2.81) [19,24]. However, although we observed this trend in our study (RR = 1.27, 95% CI 1–1.6), it was not statistically significant, perhaps related to the patient sample size.

Regarding the RDW measured after hospital admission, we found an increased mortality risk in patients with RDW > 14.5% measured at hospital discharge (RR = 2.3, 95% CI 1.89–2.81). Interestingly, final RDW levels were higher in the deceased group individuals when compared with survivors (median = 14.6 vs. 12.9, *p* < 0.001), with a difference of 1.7 in RDW%, which is considerably higher than the observed difference of 0.6 that we reported for RDW% at hospital admission. RDW measured at hospital discharge, unlike RDW measured at hospital admission, has been less exhaustively studied; Foy et al., found that those who did not survive had an average RDW increase of 1.5% during their first week of hospitalization [19], whereas survivors showed only a marginal RDW increase. This is consistent with our findings as we observed an RDW increase of 1% in non-survivors during hospitalization; on the other hand, the survivors diminished their RDW levels by 0.1%. In another study, 66% of deceased patients showed an RDW% increase over the course of hospitalization [24]. These findings suggest that an RDW increase during hospitalization could be a more important mortality predictor than the RDW at hospital admission alone. The logistic regression model showed that the combination of the final RDW value, use of mechanical ventilation, and age is a good predictor for mortality with statistical significance, and 48% of the data fitted with the regression model.

As Lee, J.J. et al. mention, the exact pathophysiology behind the association between increased RDW and adverse outcomes has yet to be elucidated [15]. Among the potential mechanisms that may explain this association are (a) stimulation to the bone marrow that may impact the RBC kinetics, resulting in a wider range of RBC size [19,25]; (b) hyperinflammatory state in certain patients with COVID-19, where the overproduction of inflammatory cytokines may influence hematopoiesis by altering the release or response to erythropoietin, thus affecting the function and structure of RBC [16]; (c) increased oxidative stress, and the release of oxygen free radicals increases [26]; (d) insufficient circulating nutrients in patients with a poor outcome may lead to an increase in RBC membrane instability, increasing the RDW [20,27]. Other physiological determinants of increased RDW are aging, black ethnicity, physical exercise, and pregnancy. RDW is useful to differentiate the etiology of different types of anemia. Anemia caused by nutritional deficiencies and chronic diseases are associated with anisocytosis and could influence a higher RDW value [28], which could be associated with the higher initial and final RDW% we observed in patients with anemia (median RDW of 14.6 and 15.4, respectively) when compared to initial and final RDW patients without anemia (median RDW of 13.2 and 13.5, respectively) [28].

In addition to the RDW, we found differences in clinical and biochemical parameters between surviving and non-surviving patients, such as age, chronic kidney disease, mechanical ventilation requirement, superinfection, initial oxygen saturation, creatinine, platelet and leukocyte count, prothrombin time, glucose, LDH, C-reactive protein (CRP), serum ferritin, and D-dimer; findings that are consistent with previous studies [29,30,31,32,33,34,35,36,37,38,39,40]. Characteristically, male sex has been associated with poor prognosis in patients having COVID-19 [33,34,36,41]; however, in our study, we were unable to find a statistically significant association (*p* = 0.754 and RR 1.02). Although these and other clinical and biochemical parameters have been used in order to predict COVID-19 severity, RDW is an easier, faster, and less expensive predictor to obtain, in comparison to other biomarkers such as CRP, D-dimer, and serum ferritin. Moreover, a recent study was unable to find any significant difference in the predictive capacity of mortality provided by the RDW (alone), SOFA, and APACHE-II [21], confirming the importance of RDW as a severity and mortality predictor in COVID-19.

Interestingly, we observed that patients under treatment with UFH showed an increased risk of death (RR = 2.59; 95% CI, 1.47–4.56); on the other hand, low molecular weight heparin (LMWH) was confirmed as a protective factor (RR = 0.5; 95% CI, 0.5–0.73). A study performed in hospitals in The Bronx, USA, that included 3625 COVID-19 patients, reported that patients with UFH prophylaxis thrice daily or full therapy were unable to decrease the risk of death (RR = 1.04 and 0.97, respectively), while LMWH/Enoxaparin was associated with decreased mortality (RR = 0.49; 95% IC, 0.32–0.73) [42]. A possible explanation for UFH treatment and the increased risk of death in our study patients could be associated to heparin-induced thrombocytopenia (HIT), a known secondary effect of UFH use. There is potential for harmful consequences since SARS-CoV-2 infection and HIT are both prothrombotic and can lead to thrombocytopenia and disseminated intravascular coagulation [43]. Due to these findings, the use of LMWH or oral anticoagulants such as apixaban could be a better option for COVID-19 patients [42].

## 5. Conclusions

We conclude that the RDW is an accessible and economical parameter that, together with other characteristics of presentation and evolution of patients with COVID-19, can be helpful in determining the prognosis. Higher RDW% at the time of hospital admission and hospital discharge were observed in non-survivors when compared to survivors. An RDW increase during hospitalization could be a more important mortality predictor than the RDW at hospital admission. The best RDW cutoff values for predicting mortality in COVID-19 patients were 13.30 and 13.70 for RDW measured at hospital admission and discharge, respectively, and the AUC was higher for RDW measured at hospital discharge.

## Figures and Tables

**Table 1:** Study group general characteristics.

	n = 323
Age, mean (SD)	55.98 (±14.16)
Death, n (%)	174 (53.9%)
Male, n (%)	211 (65.3%)
Female, n (%)	112 (34.7%)
Smoking, n (%)	22 (6.8%)
Comorbidities, n (%)	248 (76.8%)
Diabetes, n (%)	146 (45.2%)
Hypertension, n (%)	144 (44.9%)
Overweight, n (%)	90 (30.3%)
Comorbidities, n (%)	11 (3.4%)
COPD, n (%)	11 (3.4%)
Cardiopathy, n (%)	9 (2.8%)
Depression, n (%)	4 (1.2%)
Asthma, n (%)	4 (1.2%)
Cancer, n (%)	7 (2.2%)
Mechanical ventilation, n (%)	195 (60.4%)
Steroid treatment, n (%)	92 (28.8%)
Nosocomial infections, n (%)	43 (13.3)
Elevated RDW (>14.5%) at hospital admission, n (%)	55 (17.9%)
Elevated RDW (>14.5%) prior to hospital discharge, n (%)	93 (32.2%)
%SpO_2_ initial, mean (SD)	71.77(±17.4)
Number of days with mechanical ventilation, mean (SD)	9.78 (±8.74)
Hospital length of stay, mean days (SD)	10.70 (±8.42)
Overall mortality, n (%)	174 (53.87%)

COPD, chronic obstructive pulmonary disease; RDW, red blood cell distribution width; SpO_2_, oxygen saturation.

**Table 2:** Patient characteristics stratified by mortality.

	Study Group n = 323			
	Survivors (n = 149)	Non-Survivors (n = 174)	*p*-Value *	RR (95% CI)
Age	51.1 ± 14.46	60.17 ± 12.51	<**0.001**	
Male, n (%)	96 (64.4)	115 (66.1)	0.754	1.03 (0.83–1.28)
Comorbidities, n (%)	112 (75.2)	136 (78.2)	0.525	1.08 (0.84–1.39)
Diabetes, n (%)	60 (40.3)	86 (49.4)	0.099	1.18 (0.97–1.45)
Hypertension, n (%)	60 (40.3)	85 (48.9)	0.122	1.17 (0.96–1.43)
Overweight, n (%)	47 (31.5)	51 (29.3)	0.663	0.95 (0.76–1.19)
Chronic kidney disease, n (%)	1 (0.7)	10 (5.7)	**0.012**	1.73 (1.4–2.14)
Mechanical ventilation, n (%)	38 (26.2)	156 (89.7)	<**0.001**	5.7 (3.69–8.78)
Convalescent plasma, n (%)	6 (4)	2 (1.1)	0.097	0.46 (0.014–1.53)
Steroid treatment, n (%)	38 (31.2)	54 (25.9)	0.291	1.12 (0.91–1.39)
LMWH, n (%)	132 (93)	123 (75.5)	<**0.001**	0.5 (0.5–0.73)
UFH, n (%)	10 (7)	40 (24.5)	<**0.001**	2.59 (1.47–4.56)
Superinfection, n (%)	11 (7.4)	32 (18.4)	**0.004**	1.47 (1.19–1.81)
Length of stay, median (IQR)	8 (6–12)	9 (6–14)	0.080	
Days with mechanical ventilation, median (IQR)	6.5 (3–9.25)	8 (5–13)	**0.030**	
Initial SpO_2_, median (IQR),	83 (76–87)	68 (51–78)	<**0.001**	
Initial Hb, media (SD±)	14.6 ± 2.27	14.08 ± 2.20	**0.044**	
Initial Hb < 12 g/dL, n (%); n = 314	15 (10.2)	22 (13.2)	0.415	1.14 (0.85–1.52)
Initial MCV, mean (SD±)	89.88 ± 6.53	91.33 ± 6.87	0.057	
Initial MCV > 100 fL, n = 314	6 (4.1)	11 (6.6)	0.328	1.23 (0.85–1.78)
Initial RDW, median (IQR) n = 308	13 (12.3–13.7)	13.6 (12.95–14.4)	<**0.001**	
Initial RDW > 14.5%, n (%)	19 (13.3)	35 (21.2)	0.068	1.27 (1–1.6)
Final RDW, median (IQR)	12.9 (12.2–13.5)	14.6 (12.67–15.6)	<**0.001**	
Final RDW > 14.5, n (%); n = 289	13 (9.6)	81 (52.6)	<**0.001**	2.3 (1.89–2.81)
Initial leukocyte count, median (IQR)	6.93 (5.13–8.37)	11.7 (8.36–16.20)	<**0.001**	
Initial leukocyte count > 11 × 10^3^/μL, n (%); n = 312	41 (28.1)	94 (56.6)	<**0.001**	1.72 (1.39–2.11)
Initial glucose, median (IQR)	126 (103–184)	173 (115–272)	<**0.001**	
Initial glucose > 110 mg/dL, n (%); n = 308	89 (62.7)	133 (80.1)	<**0.001**	1.56 (1.17–2.08)
Initial creatinine, median (IQR)	0.80 (0.6–0.9)	1 (0.7–1.5)	<**0.001**	
Initial creatinine > 1.3 μmol/L, n (%); n = 312	7 (4.8)	52 (31.1)	<**0.001**	1.94 (1.64–2.28)
Initial LDH, median (IQR)	368 (301–524)	515 (413–648)	<**0.001**	
Initial LDH > 245 U/L; n = 209	87 (96.5)	111 (92.6)	0.201	1.54 (0.69–3.4)
Initial CRP, media (SD±)	178.29 ± 117.72	216.92 ± 122.18	**0.006**	
Initial CRP > 30 mg/L, n (%); n = 292	127 (92)	149 (96.8)	0.077	1.73 (0.83–3.6)
Initial serum ferritin, median (IQR)	581 (244–1020)	805 (511–1247)	0.070	
Initial ferritin > 300 ng/mL; n (%) n = 74	26 (45.6)	31 (54.4)	**0.025**	2.3 (0.95–5.6)
Initial D-dimer, median (IQR)	369 (268–727)	637 (337–1543)	<**0.001**	
Initial D-dimer > 500 ng/mL, n (%); n = 275	46 (34.8)	75 (59.4)	<**0.001**	1.61 (1.27–2.04)
Initial PT, median (IQR)	13.1 (12.3–14-1)	13.6 (12.4–14.8)	**0.020**	
Initial PT > 16 s, n (%); n = 307	5 (3.5)	20 (12.2)	**0.005**	1.57 (1.25–1.97)

LMWH, low molecular weight heparins; UFH, unfractionated heparin; SpO_2_, oxygen saturation; Hb, hemoglobin; MCV, mean corpuscular volume; RDW, red blood cell distribution width; LDH, lactate dehydrogenase; CPR, C-reactive-protein; PT, prothrombin time. * Statistical significance was calculated using a 2-sided *t*-test for means, Mann–Whitney U test for quantitative variables with non-parametric distribution, and χ^2^ test for percentages.

**Table 3:** Comparison of initial and final RDW% among COVID-19 survivors and non-survivors.

	Initial RDW%	Final RDW%	Difference (Δ)	*p*-Value *
Survivors, median (IQR)	13 (12.3–13.7)	12.9 (12.2–13.5)	−0.1	0.005
Non-survivors, median (IQR)	13.6 (12.94–14.4)	14.6 (12.67–15.6)	+1.0	<0.001

RDW, red blood cell distribution width. * Wilcoxon signed-rank test.

**Table 4:** Results of the receiver operating characteristic (ROC) curve, diagnostic utility analysis, and the logistic regression analysis for mortality.

Parameter	Area under the ROC Curve (AUC)	95% CI	Cutoff	Sensitivity	Specificity
Initial RDW	0.655	0.585–0.715	≥13.30	0.621	0.644
Final RDW	0.830	0.773–0.873	≥13.70	0.758	0.788
**Independent variable**	**Regression coefficient**	**Odds Ratio (95% CI)**	**Wald *p*-value**	**Model R^2^**	**AUC** **(95% CI)**
Intercept	−14.501	0.0 (0.00–0.001)	<0.001	0.483	0.917 (0.832–0.960)
Final RDW	0.913	2.49 (1.585–3.916)	<0.001		
Mechanical ventilation	−2.558	0.08 (0.026–0.230)	<0.001		
Age	0.052	1.05 (1.014–1.094)	0.007		

## Data Availability

All relevant data are within the paper.
